# py_ped_sim: a flexible forward pedigree and genetic simulator for complex family pedigree analysis

**DOI:** 10.1186/s12859-025-06142-z

**Published:** 2025-05-07

**Authors:** Miguel Guardado, Cynthia Perez, Sthen Campana, Berenice Chavez Rojas, Joaquín Magaña, Shalom Jackson, Emily Samperio, Selena Hernandez, Kaela Syas, Ryan D. Hernandez, Elena I. Zavala, Rori V. Rohlfs

**Affiliations:** 1 Department of Mathematics, San Francisco State University, San Francisco, CA 94132 USA; 2 Biological and Medical Informatics Graduate Program, University of California San Francisco, San Francisco, CA 94158 USA; 3 Bioengineering and Therapeutic Sciences, University of California, San Francisco, CA 94134 USA; 4 Department of Data Science, University of Oregon, Eugene, OR 97403 USA; 5 Department of Biology, San Francisco State University, San Francisco, CA 94132 USA; 6 Department of Molecular and Cell Biology, University of California, Berkeley, Berkeley, CA 94720 USA

**Keywords:** Forward-time simulations, Pedigree, Genetics, Kinship, Family, Generation, Inheritance, Python

## Abstract

**Background:**

Large-scale family pedigrees are commonly used across medical, evolutionary, and forensic genetics. These pedigrees are tools for identifying genetic disorders, tracking evolutionary patterns, and establishing familial relationships via forensic genetic identification. However, there is a lack of software to accurately simulate different pedigree structures along with genomes corresponding to those individuals in a family pedigree. This limits simulation-based evaluations of methods that use pedigrees.

**Results:**

We have developed a python command-line-based tool called py_ped_sim that facilitates the simulation of pedigree structures and the genomes of individuals in a pedigree. py_ped_sim represents pedigrees as directed acyclic graphs, enabling conversion between standard pedigree formats and integration with the forward population genetic simulator, SLiM. Notably, py_ped_sim allows the simulation of varying numbers of offspring for a set of parents, with the capacity to shift the distribution of sibship sizes over generations. We additionally add simulations for events of misattributed paternity, which offers a way to simulate half-sibling relationships, and simulations to extend the breadth of a family pedigree. We validated the accuracy of both our genome simulator and pedigree simulator. We show that we can simulate genomes onto family pedigrees with levels of expected kinship.

**Conclusions:**

py_ped_sim is a user-friendly and open-source solution for simulating pedigree structures and conducting pedigree genome simulations. It empowers medical, forensic, and evolutionary genetics researchers to gain deeper insights into the dynamics of genetic inheritance and relatedness within families.

**Supplementary Information:**

The online version contains supplementary material available at 10.1186/s12859-025-06142-z.

## Background

Genetic pedigrees are essential for studying medical, evolutionary, and forensic genetic inheritance. Pedigrees have been instrumental in understanding disease prevalence within families and populations and guiding genetic counseling and management strategies. They have been used to unravel the genetic influence of many complex traits, such as psychiatric disorders and neurodegenerative diseases, via family-based studies [1–3]. Additionally, pedigrees help give us insight into rare variants acting on disease [2, 4–6]. It has been shown that rare variant sharing is related to individuals'demographic and familial history [7], emphasizing the need to understand how rare variations are transmitted through families. In evolutionary genetics, family pedigree studies provide insights into population dynamics [8–10], the heritability of traits [11–13], and natural selection [14, 15]. The emerging forensic practice of investigative genetic genealogy (IGG) relies heavily on pedigree analysis to connect genetic relatives [16–18]. By constructing pedigrees and tracing familial relationships, investigators can identify potential suspects and narrow down a pool of individuals who may be related to unidentified remains and DNA evidence found at a crime scene [18, 19].

In order to understand how traits are passed through pedigrees, genetic simulations are leveraged to generate individual genomes onto pedigree structures. These simulators have been used to study the genetic inheritance of genetic traits [20], test kinship methods [21], and help understand how migration and pedigree relationships result in patterns of genetic variation today [22]. Initial tools for these simulations were intended for relatively few loci or small pedigrees using gene-dropping [23–26] and have been widely employed within the research community. GENLIB [27] presented a more computationally efficient method, allowing for multi-locus, large family simulations for forward gene-dropping pedigree simulation. ped-sim [28] simulates full genomes forward in time based on a defined pedigree using sex-specific genetic maps crossover interference, and user-defined missingness and error rates. The backward-simulator MSPrime [22, 29] recently added a feature to simulate genomes on fixed pedigrees, but is limited to only complete pedigrees or individuals who have both parents known and identified. SLiM, a widely used forward genetic simulator known for its highly scriptable and flexible framework, also can simulate genomes based on fixed pedigree structures [30, 31]. While SLiM has a feature to simulate genomes based on fixed family pedigrees, it requires prior knowledge of the family structure, such as the list of founders in the pedigree and the generation in which descendants are created. Having to identify this information manually causes a barrier to performing genomic simulations on large sets of family pedigrees in an automated manner.

Pedigree structures play a crucial role in shaping a family's genetic architecture. Currently, available pedigree simulators do not include the ability to vary the rates of sibship across generations or model unknown half relationships. Some half-relationships are known, but they can also result from misattributed genetic paternity (MAP), with the latest rate measured at 1.9% in human offspring.[32–34]. Additionally, MAP rates vary across different countries and groups [34]. Modeling these events into the pedigree structure can help us understand inconsistency within family pedigrees and the impact that can have on the analysis of genetic kinship. Additionally, sibship size per generation has decreased in the last century and has been shown to vary dramatically across different countries [35]. This becomes an important parameter to consider in modeling a pedigree since the rate of sibship will impact the genetic inheritance and the resulting genetic variation of a population. A pedigree simulator that can factor in real-world data for sibship rates and model MAP can better reflect the complexities of human populations and improve our understanding of how demographic history impacts genetic variation. Currently, no pedigree structure simulator exists to the author's knowledge with the ability to model dynamic pedigrees with changing sibship sizes or MAP rates with or without real-world data.

**Fig. 1:**
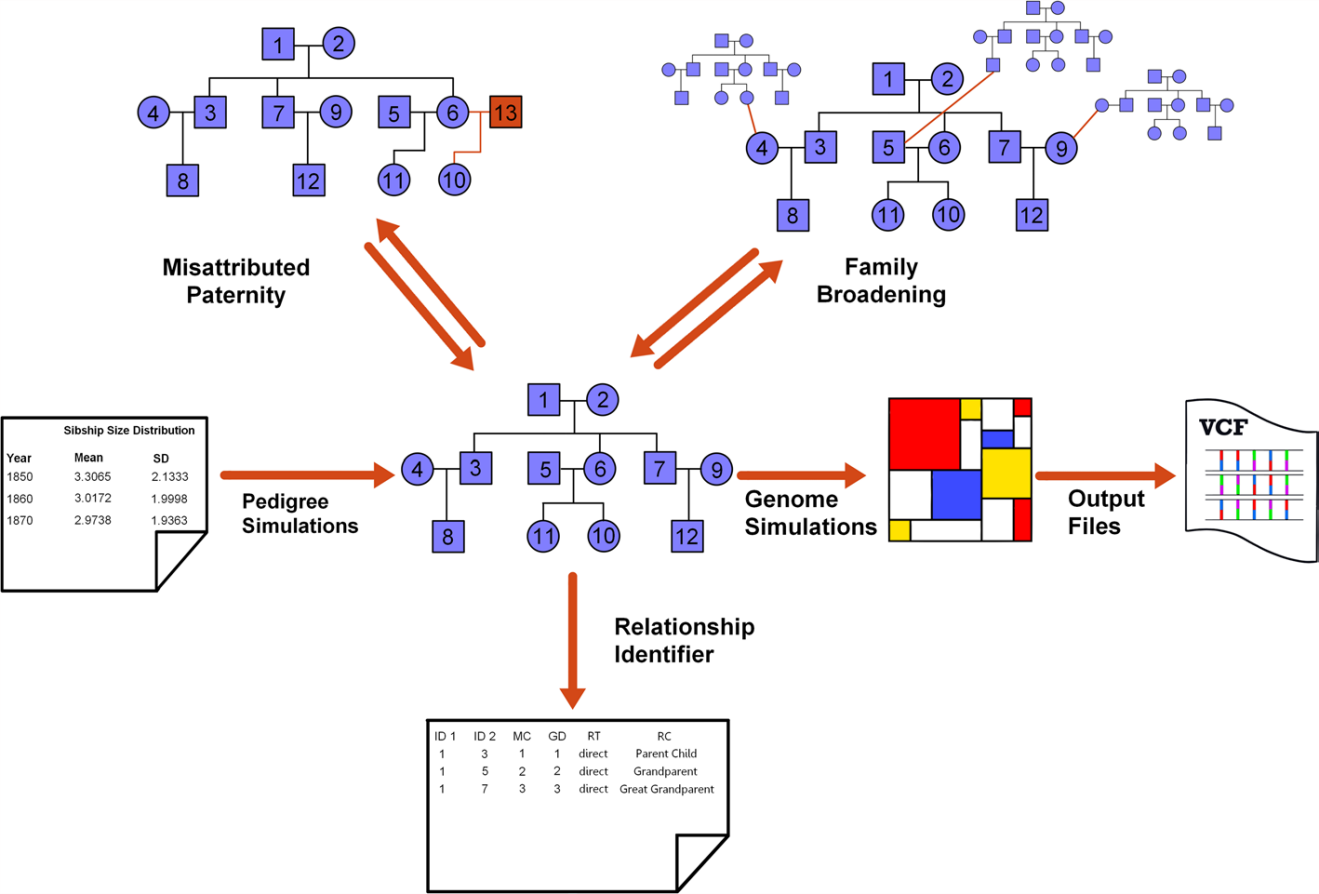
Visual abstract illustrating the primary features of the py_ped_sim software. Our software comes with the ability to (Pedigree Simulations, 1) simulate pedigrees with non-uniform sibship sizes, (Misattributed Paternity, 2) simulate events of MAP onto existing pedigree structures, (Family Broadening, 3) simulate family broadening, extending the horizontal breadth of the family, (Genome Simulations, 4) simulate genomes onto pedigrees using SLiM, (Relationship Identifier, 5) identify all genetic relationships within the family pedigree

Here, we present py_ped_sim, a command line tool in python designed to integrate the simulation of dynamic pedigrees and genomes. Our software incorporates five key features (Fig. 1a): (1) the simulation of varied genetic pedigree structures based on sibship sizes over time, (2) the simulation of misattributed paternity events within family pedigrees, (3) the simulation of genomes based on a pedigree, (4) the ability to increase the breadth of a family pedigree, and (5) the identification of pairwise relationships within a given family pedigree (Fig. 1a). To enable efficient genetic simulations on complex or incomplete pedigrees, we have developed a wrapper for SLiM that allows users to input varied pedigree data. To implement these features, we represent family pedigrees as directed acyclic graphs and allow users easy conversion between traditional pedigree data formats and directed graphs. We demonstrate that py_ped_sim accurately generates genomes on family structures with expected genetic relationships and report validations for our pedigree simulation features. Notably, our pedigree simulator has non-uniform rates of sibship across generations, MAP simulations, and the ability to extend the breadth of the family.

## Implementation

### Definitions

- DAG—Directed Acyclic Graph. Where individuals are nodes and edges are parent–child relationships.
- Descendant—offspring; an individual that has at least one known parent.
- Generation Tick—Our definition of generation tick is the same as that in SLiM. Generations are a biological unit of time, relating to things like an organism's lifespan. Root founders are defined with a generation tick of one, while their children are in generation 2, and so forth.
- Root Founder—A node that is connected to all lower-level nodes. The root founder of the pedigree will have a generation tick of one.
- Explicit Founders—Individuals specified in the pedigree with no known ancestors.
- Implicit Founders—Missing parents for individuals in the pedigree with only one known ancestor.
- Pedigree formats
  - Traditional pedigree formal (.ped/.fam): This data representation of a family is traditionally used where the columns are represented as [FID, IID, P1, P2, Sex, Phenotype]
  - Networkx-based pedigree (.nx): Family pedigree based on a directed acyclic graph implemented in networkx. The files are two-column files; the first column is the parent and the second is a descendant.
  - Slim-Readable pedigree (_slim_pedigree.txt): Family pedigree file that is readable by SLiM. Similar to a.ped file but includes and orders the generation in which descendants are created [Gen, IID, P1, P2].

### Dependencies and versions

This software is developed in python (version 3.88), using SLiM (version 4.0), and bcftools (version 1.8). All user interaction with the software is through a python front end. All software dependencies can be accessed via a conda virtual environment.

### Data structure of family pedigree

We represent family pedigrees as directed acyclic graphs (DAGs) using the networkx package in python 3.8 [36]. The DAG is comprised of nodes and edges, where nodes represent individuals and directed edges indicate parent-to-child genetic transmission (Fig. 1b, networkx pedigree schematic). Because an individual cannot be their own genetic ancestor in a sexual reproductive system, these graphs are acyclic, meaning the directed path of the edges never forms a loop.

### Pedigree simulator

Py_ped_sim forward simulates pedigrees with the number of offspring per pair of genetic parents taken from user-provided data on sibship sizes. The novelty of our approach is the ability to vary the sibship distributions used across generations. We draw sibship sizes from normal distribution based on the user-provided generation, mean, and standard deviation. The user will include a CSV file on each generation tick and their mean and standard deviation for sibship size. For convenience, we provide a default sibship distribution file with data from the United States Census obtained via IPUMs [37].

We utilize a depth-first recursive approach to simulate children until the last generation is reached. Users specify the number of generations in the pedigree via the number of generation ID requested. The number of offspring is drawn from user-provided sibship sizes for each set of parents for the generation number. Our software additionally simulates an individual's sex and keeps track of the generation time in which individuals are created. We define the sex of an individual by whether a fertile individual produces a sperm (XY) or an egg (XX). It is important to note that we don't simulate sex chromosomes in our simulation framework. We simulate the sex of an individual via drawing from a Bernoulli distribution to determine the sex of the first parent and set the sex of the second parent to the complement.

The output of py_ped_sim is a pedigree in networkx format, in addition to a profile file that contains the sex and generation tick of everyone simulated. py_ped_sim pedigrees are output as DAGs via networkx format files (.nx).

### Misattributed paternity simulator

Modeling MAP events can offer another way to simulate more dynamic family pedigrees and investigate how MAP can impact downstream conclusions. We have incorporated a feature for simulating misattributed paternity events on existing family pedigrees. A complimentary benefit of introducing this to family pedigree simulations is adding genetic half relationships.

Our simulations of MAP events involve a three-step process: (1) selecting individuals that could possibly have MAP, (2) randomly determining if each of those individuals has MAP, and (3) determining if the new genetic father will come from within the family or through the introduction of a new individual in the family pedigree. First, to select individuals who could have a MAP event, we identify all individuals with genetic parents specified in the pedigree. Second, for each of those individuals, we draw a binary variable via a Bernoulli distribution with a user-provided parameter to determine if the individual has MAP. Third, when a MAP event occurs, we determine if the new genetic parent will be an existing individual in the pedigree, or if we will create a new individual. To do so, again, we will sample from a Bernoulli distribution with a user-specified parameter. In the case when the parent is an already-existing individual, we sample randomly from males of the same generation tick as the male being replaced. This can potentially introduce consanguinity in the event the new father assigns the mother’s brother or cousin. The user can eliminate the change of consanguinity by setting the within-fam probability parameter to zero. When the genetic father is from outside the family, py_ped_sim will generate a new individual to perform the MAP.

### Genome simulator

To initiate the genomic simulations, users specify the genetic pedigree structure, along with a genomic file (.vcf) for initializing the founders of the family. The user can specify the mutation and recombination rates for the simulations, with default values having a mutation rate of 1e-8 and a recombination rate of 1e-7. Users also have the flexibility to input a recombination map to vary the recombination rate across the genome they are simulating. We provide two methods for initializing the founder genomes based on a user-provided vcf file. The first method randomly assigns each founder to an individual in the vcf file. Alternatively, users can provide an additional text file that assigns specific founders to corresponding IDs in the vcf file, offering more control over the initialization process. The founder genome can be simulated as a supplementary feature, which is particularly useful if an appropriate genome dataset for founder initialization is unavailable. This feature will generate genomes using the msprime sim_ancestry feature, where the user can specify the number of individuals to have in the population, the desired length of the genome, and the number of individuals to sample. Finally, users can perform nucleotide-specific simulations, which allows the exact positions of the founder’s genomes to be maintained, allowing the user to compare their simulations with external genetic resources. To use this feature, users provide a fasta file of the reference sequence of the genomes used to initialize the founders.

We introduce a feature that uses SLiM to simulate genomic variation on family pedigrees. This feature is a wrapper script that extracts features of the family pedigrees that SLiM requires for genomic simulations. Both explicit and implicit founders are to be identified alongside generation numbers for all descendants created. py_ped_sim represents the pedigree as a DAG; in that format, we can extract information on descendants'generation tick and founders of the pedigree. The output is a vcf file of the simulated genomes for everyone in the pedigree. The vcf does not include the genomes of implicit founders, that is, parents not specified in the original pedigree.

Identifying founders in simulations is crucial to supplying genomes for individuals without identified parents in the pedigree, establishing the genetic variation for individuals for all non-descendants. We identify individuals with no predecessors (explicit founders) and one known predecessor (implicit founder events). We start by identifying the family's root founders and assigning them a generation tick of one. The generation ticks of descendants are then assigned based on their shortest path to root founders. For individuals not directly descended from a root founder, we calculate the generational gap between the individual in question and a root founder’s descendant and use the difference to determine the generation number for the individual in question. In the event of consanguinity, when individuals have more than one path to the root founder, we ensure the child’s generation tick number is after their parent's.

### Pairwise relationships identifier

**Fig. 2:**
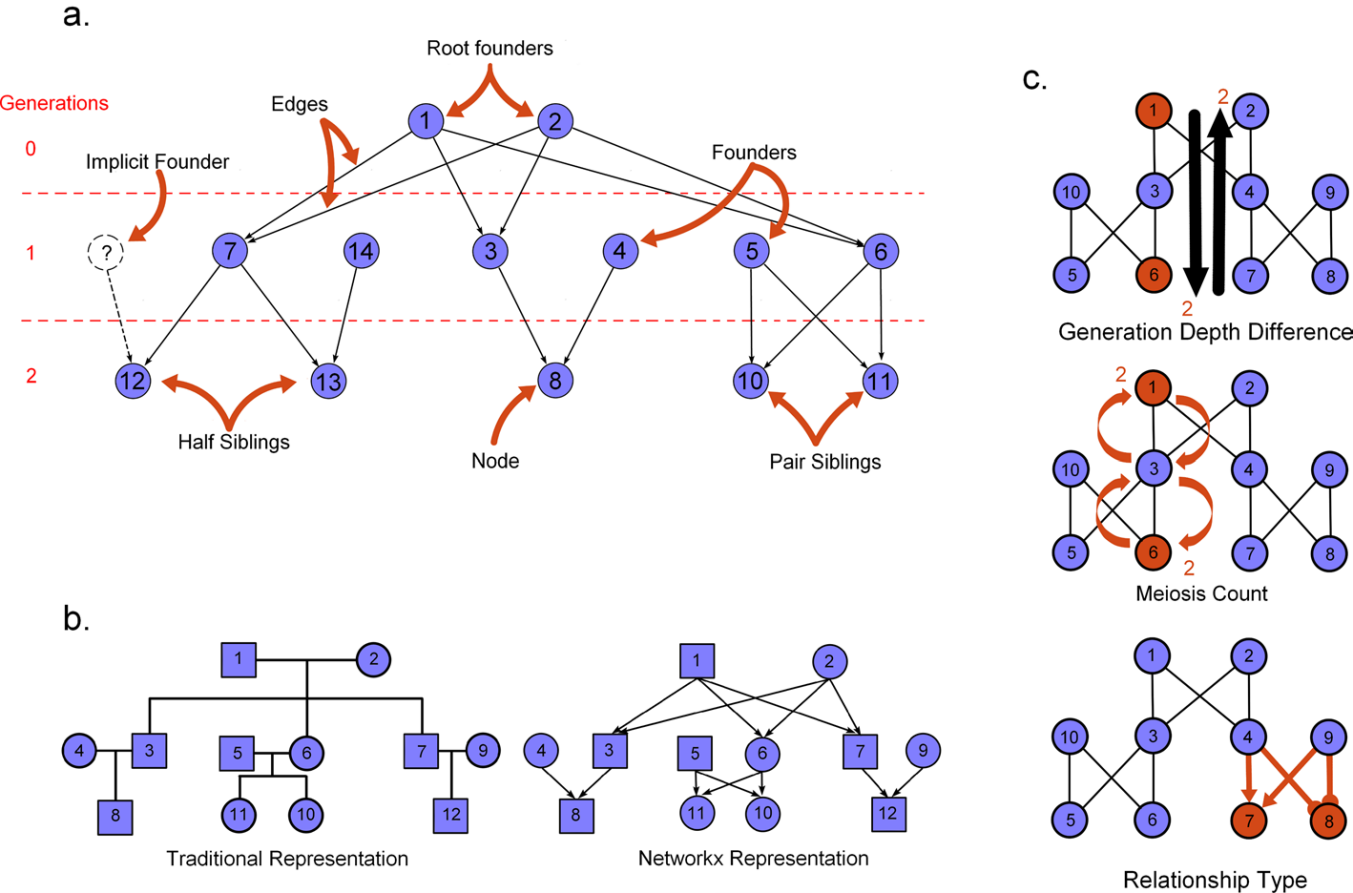
Schematic overview of the pedigree definitions used in py_ped_sim. **A** General definitions of family pedigree terms (Table 1). **B** Comparison of traditionally represented pedigree vs networkx-represented pedigrees. Networkx-represented pedigrees follow a directed acyclic graph where nodes represent individuals and directed edges identify parent–child relationships. **C** Overview of genetic relationship metrics: generation depth, meiosis count, and relationship type. Examples are visualized for each relationship metric for an avuncular relationship

We introduce a feature to provide a generalizable representation of genetic relationships for all pairs of individuals inside a pedigree. For some analyses, it is useful to isolate particular genetic relationships in large pedigrees where manual relationship identification is not feasible. Our software can quantify genetic relationships between pairs of individuals in a pedigree, defined by three deterministic metrics: meiotic distance (MD), the generation depth difference (GDD), and the genetic relationship type (GRT) (Fig. 2c). These three statistics help to code pairwise genetic relationships into genetic relationship categories (siblings, half-first cousins, etc.).

The meiotic distance between two individuals describes how many meioses separate them. An avuncular (e.g., aunt-nephew) relationship has an MC of three (Fig. 2c). Half-siblings have an MC of two since they connect via one shared parent. If a direct genetic relationship exists, that is, a relationship where one individual is a direct descendant of the other (parent, grandparent, great-grandparent, etc.), the MD is the length of the shortest path between the individuals. Otherwise, the MD is the sum of the distances between each individual and their most recent common ancestor.

GRT describes if two individuals are full genetic relatives (two shared ancestors), half genetic relatives (one shared ancestor), direct genetic relatives (defined above), or not determinable (NA). Let's take the example of an avuncular relationship. If there are two common ancestors for a child and their uncle/aunt, i.e., the uncle/aunt is a full sibling of the child’s parent, so they are both descended from both of the child’s grandparents, then the relationship is classified as a full relationship (Fig. 2c). To determine GRT, we find all the shortest paths between two nodes. To determine direct and half relatives, we count the number of shortest paths by which two individuals are connected. If there is one path, they're half-genetic relatives; if there are two paths, they are full genetic relatives.

Finally, the GDD between two individuals is the number of generations separating them. Using the same examples of avuncular and half-sibling relationships, we see GDDs of 1 and 0, respectively (Fig. 2c). GDD is the shortest path between the two nodes for individuals with a direct genetic connection. Otherwise, GDD equals the absolute value of the difference between the shortest path lengths from the two individuals to their shared ancestor.

### Family broadening

Family Broadening (FB) simulates a genetic pedigree context for non-root founders to expand the breadth of the pedigree and capture otherwise missing relatives. The process begins with a central family, either specified by the user or simulated using py_ped_sim. Non-root founders, identified as individuals with no ancestors who are not in generation zero, are used for family expansion. For each non-root founder, a pedigree is simulated and an individual with the same sex and in the same generation as the non-root founder is replaced by the non-root founder, connecting the new simulated pedigree to the central pedigree, thus greatly increasing the number of relationships for descendants of the non-root founders. Pedigrees simulated to attach to the central pedigree are generated using py_ped_sim, with IDs incrementally assigned based on the maximum from previous iterations. The final outputs will include updated profile files for the sex and generation tick for the new individuals added to the family pedigree.

## Results

### Validation of simulated pedigree structures

Our software can simulate pedigrees based on user-specified sibship size distributions for each generation. We used py_ped_sim to simulate 10,000 families across five generations to assess its ability to simulate non-uniform sibship sizes. We used sibship size estimates from IPUMs [37] to simulate our pedigrees, specifically for census years spanning from 1850 to 1970 (1850, 1880, 1910, 1940, 1970) (Table 1). We estimated the observed mean and standard deviation of sibship size for the 10,000 simulated pedigrees across each generation (Table 1). The observed simulated sibship sizes are closely aligned with the sibship size means and standard deviations provided as parameters to the simulation. These findings confirm py_ped_sim’s ability to simulate pedigrees with changing sibship sizes across successive generations.

**Table 1:** Validation results of py_ped_sim’s pedigree structure simulator. We present the true distributions of specified sibship sizes alongside the observed distributions generated by py_ped_sim

	Specified mean	Specified SD	Num sibships simulated	Observed mean	Observed SD
Gen 1850	3.31	2.13	10,000	3.32	2.03
Gen 1880	3.02	1.95	33,169	3.04	1.86
Gen 1910	2.72	1.85	100,688	2.78	1.77
Gen 1940	2.35	1.70	280,343	2.41	1.61
Gen 1970	2.30	1.43	675,309	2.33	1.40

**Fig. 3:**
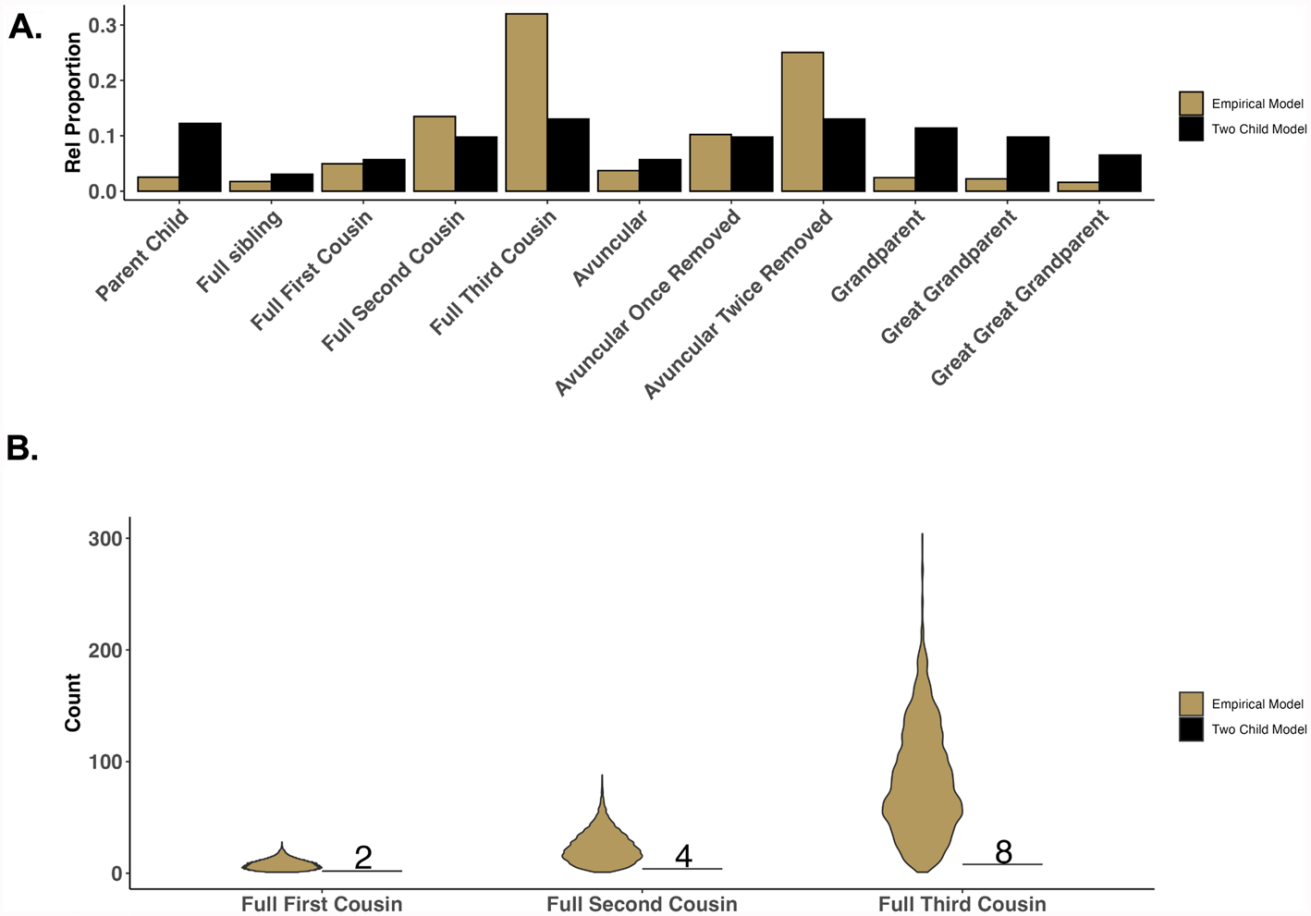
Comparison of individual-level variation in the degree of cousin relationships across two simulated family pedigree models, a two-kid model (black), and an empirical model (gold)

To demonstrate the impact differing sibship size models may have, we compared a two-kid model and an empirical sibship size model. The former assumes each pair of parents has two children, while the latter draws distributions of sibship sizes from the above-mentioned IPUMs census data. Four-generation pedigrees were simulated under both models, with 1,000 pedigrees simulated under the empirical sibship size model to provide a distribution of pedigrees created with our model. We identified all genetic relationships using py_ped_sim’s pairwise relationship identification feature*.* Figure 3 compares the distribution of the number of cousins between the two models, showing many more cousins under the empirical sibship size model (Fig. 3a). The number of cousin relationships per individual varies substantially, particularly among more distant cousins (Fig. 3b). Overall, py_ped_sim demonstrates the capacity to generate pedigrees with a more dynamic representation of distant relationships by offering user-provided varying sibship sizes over generations.

**Fig. 4:**
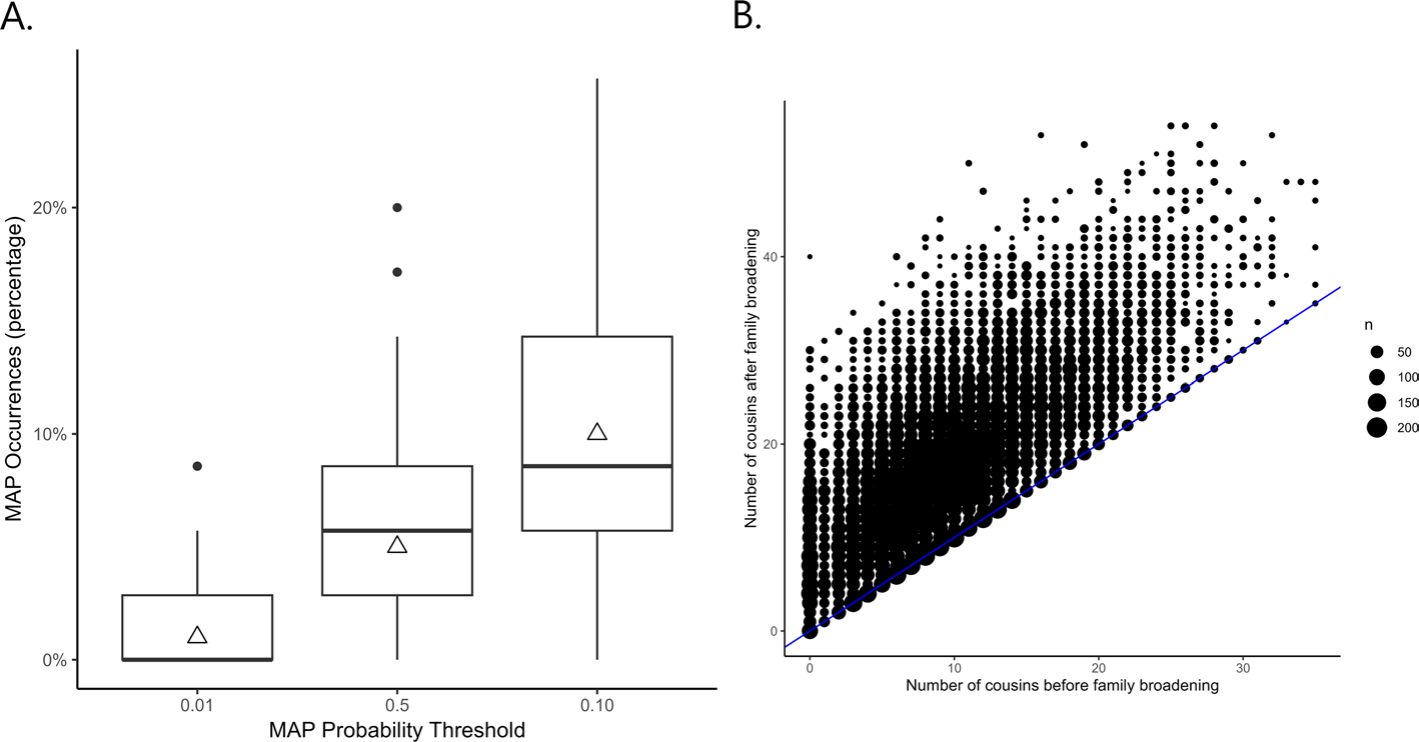
Validation of supplemental pedigree simulators, Misattributed Paternity (MAP), and Family Broadening (FB). **A** The number of MAP events across the MAP parameter values. The box plots show the total number of MAP events for each of the 1,000 simulations. The triangles indicate the expected number of MAP events for each MAP parameter value. **B** The number of first-cousin relationships inside a family pedigree before and after 1000 family broadening simulations. The size of the dots represents the density of first cousin relationships that exist for individuals before and after family broadening

To validate py_ped_sim’s misattributed paternity (MAP) feature, we simulated 1,000 MAP events for a single family using three MAP probabilities: 0.01, 0.05, and 0.10. The specified pedigree consisted of 46 individuals with no half relationships, with 35 eligible to undergo MAP events. As the MAP probability increases, the number of half relationships created also increases, approximating the expected number of MAP events (Fig. 4a). This validation demonstrates py_ped_sim’s ability to simulate MAP events within family pedigrees.

Finally, we validate the ability to extend the breadth of a family using py_ped_sim’s family broadening (FB) feature. We simulated family broadening 1000 times on a singular simulated family pedigree. Pairwise relationships were identified using our software before and after the family broadening was performed to determine the number of cousin relationships our simulation created. Figure 4b counts the number of cousins an individual has before and after the FB was performed. The blue line represents no increase in the number of cousins for an individual. Most individuals in the original family pedigree have a dramatic increase in the number of cousins after the family broadening was performed, with a few individuals having no increase in the number of relationships found. This validation shows that family broadening can increase the breadth of the family, thereby creating more cousin relationships.

### Validation of genetic relationships via estimated kinship

**Fig. 5:**
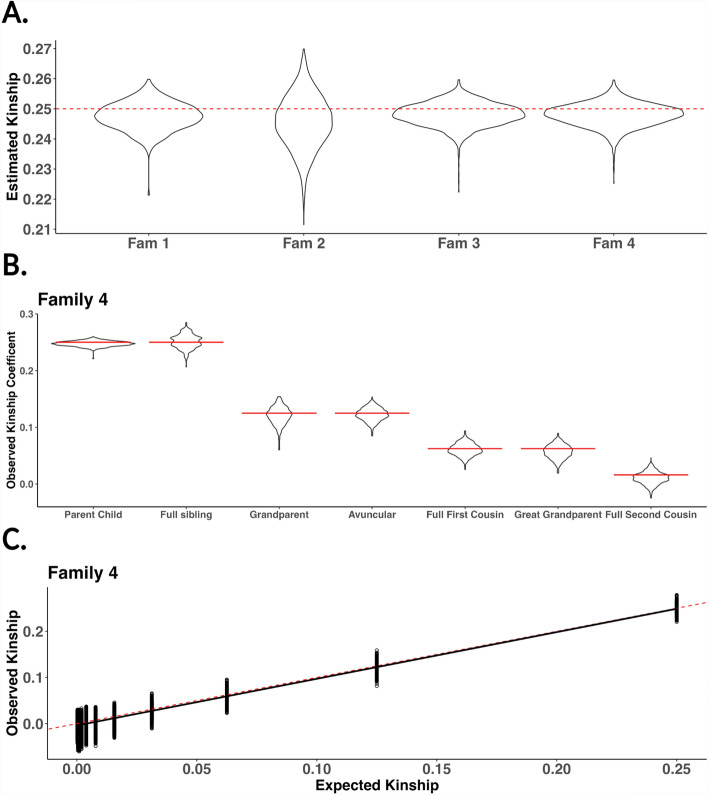
Estimated kinship for four simulated families using py_ped_sim. **A** Distributions of observed kinship for all parent–child relationships for the six families simulated. **B** For Family 4, the largest family, expected (red line) vs. observed kinship for seven different relationships found in the family. **C** Scatter plot of observed vs expected kinship for all relationships found in Family 4. The solid black line is a regression between estimated and expected kinship. The red dashed line is a regression of the expected kinship across each relationship

To test the validity of py_ped_sim’s SLiM wrapper to simulate genomes onto pedigrees, we simulated genomes for individuals in four pedigrees (Table 2) and estimated pairwise kinship coefficients among members of these families (Fig. 5). The four families were simulated to increase in size for both the number of founders needed to initialize and the total size of the family. Five generation pedigrees were simulated with py_ped_sim using sibship distribution parameters from IPUMs [37]. Genomic simulations on the pedigrees were performed with a constant recombination rate of 1e-6 and a mutation rate of 1e-7. We selected a recombination rate of 1e-6 based on a preliminary genomic simulation analysis of the first family (Supplemental Figure S1). Using the same family and set of founders, we simulated genomes under three different recombination rates (1e-6, 1e-7, and 1e-8). Our analysis showed no statistically significant differences in the distribution of parent–child kinship estimates across these rates (Figure S1A). However, we observed greater variability in kinship estimates for more distant genetic relationships, which can be observed when simulating lower recombination rates on a single chromosome (Figure S1B-D). We performed pairwise kinship using KING [38] and hierfstat [39, 40], we focused on reporting results with KING due to their ability to provide stable kinship estimates for parent–child relationships (Supplemental Figure S2). We retain kinship results using hierfstat for parent–child relationships. Founders were initialized using empirical data from the 1000 Genomes Consortium using unrelated individuals from the African superpopulation group (454 individuals) [41]. Using hap-ibd to estimate pairwise IBD [42], we define individuals as unrelated if they have IBD sharing less than 1.5%. Lastly, we identified all genetic relationships with the py_ped_sim pairwise relationships identifier feature.

**Table 2:** Six simulated families used to simulate genomes using py_ped_sim

	Number of founders	Total family size
Fam 1	53	221
Fam 2	180	724
Fam 3	236	929
Fam 4	419	1661

For four families, 95% of parent–child relationships have kinship estimates that fall within the range of 0.24–0.26 (Fig. 5a) (see results for hierfstat in Supplemental Figure S2). Overall, we show that we can simulate genomes onto family pedigrees with expected levels of kinship between parent–child relationships, which is the crux of what is being simulated in SLiM. Using the largest family simulated, we then explored the observed kinship for more distant genetic relationships (Family 4). We explored genetic relationships for the fourth family since we observed similar distributions of kinship for parent–child relationships in all the families simulated. The average kinship estimates for seven relationships converged closely to their expected values in the pedigree (Fig. 5b) (Table 3). Finally, we looked at the observed vs expected kinship within all genetic relatives inside the pedigree. We estimated the expected kinship with KinInbCoef. We found a strong correlation (R^2^ = 0.87) between observed and expected kinship for the fourth family (Fig. 5c). These results demonstrate py_ped_sim’s capability to simulate genomes onto family pedigrees with expected levels of kinship across various genetic relationships with expected genetic kinship. This validation also extends to the SLiM simulator, as py_ped_sim is a wrapper that uses SLiM for genomic simulations.

**Table 3:** Expected and observed kinship statistics of the simulated genomes for Family Six across seven different relationship statistics (Fig. 5b)

Relationship	Expected kinship	Observed kinship mean	Observed kinship standard deviation	Observed kinship median	Observed kinship IQR
Parent–Child	0.250	0.248	0.004	0.248	0.004
Sibling	0.250	0.249	0.009	0.249	0.013
Grandparent	0.125	0.122	0.010	0.122	0.014
Avuncular	0.125	0.122	0.008	0.122	0.011
1 st Cousin	0.063	0.059	0.008	0.060	0.011
Great grandparent	0.063	0.059	0.011	0.059	0.014
2nd cousin	0.016	0.012	0.009	0.012	0.012

Additionally, we conducted a validation experiment to simulate genomes using genetic recombination maps, to demonstrate that segment-based kinship can be simulated at expected levels of relatedness. We used a recombination map estimated by Halldorsson et al. 2019 [43] to simulate chromosomes 1–22 for a small family of twelve individuals, we estimated segment-based kinship using hap-ibd [42]. Supplemental Figure S3 shows the total percentage of the genome shared as IBD for each pair of individuals in the family. Parent–child and sibling pairs share around 50% of their genomes, while more distant relatives fall within the expected ranges of shared genomic segments. These results confirm that our approach can successfully simulate genomes with appropriate kinship levels using segment-based methods.

Finally, we conducted a time assessment test of our genome simulator on a 12-generation family pedigree with 3,811 individuals and 494 founders from Familinx [44]. Genomes were initialized using neutral burn-in simulations from MSprime for both a 100 Kb and 10 Mb region, run on a high-performance computer with 10 GB of memory. The 100 Kb region completed almost instantly (CPU ~ 1 s), while the 10 Mb region took approximately three hours and 24 min.

### Kinship estimations across various assumptions of pedigree structure

Next, we want to consider how expected vs observed kinship will change across various pedigree structures. We simulate genomes onto five pedigree structures: an empirical family obtained from Familinx, a simulated two-kid model, a census-based simulated family, and two simulated families with varying levels of misattributed paternity (MAP = 0.01, 0.05) (Table 4). Familinx is a crowdsourced genealogical dataset with large empirical pedigrees, including families with consanguinity [44]. Similar to our previous experiment, we used a mutation rate of 1e−7 and a recombination rate of 1e-6. We used the same individuals from the African superpopulation group from the 1000 genomes consortium. For the five pedigree structures considered, we kept the size of the family (1600–2000 individuals) and number of founders initiated (402–433) within a similar range of each other (Table 4). The only exception is the two-kid simulated family, having a pedigree of 766 individuals and a founder size of 256 individuals. This is due to the growth in the deterministic two-kid family pedigree model when adding another generation, creating more founders than can be initialized by the unrelated individuals from the African superpopulation.

**Table 4:** Five families used to simulate genomes using our genome simulator software. CS stands for census-simulated, with MAP being misattributed paternity events

	Pedigree structure type	Number of founders	Total family size
Fam 1	Empirical	402	2018
Fam 2	2-Kid Simulated	256	766
Fam 3	Census Simulated (CS)	419	1661
Fam 4	CS + MAP (0.01)	421	1663
Fam 5	CS + MAP (0.01)	433	1675

**Fig. 6:**
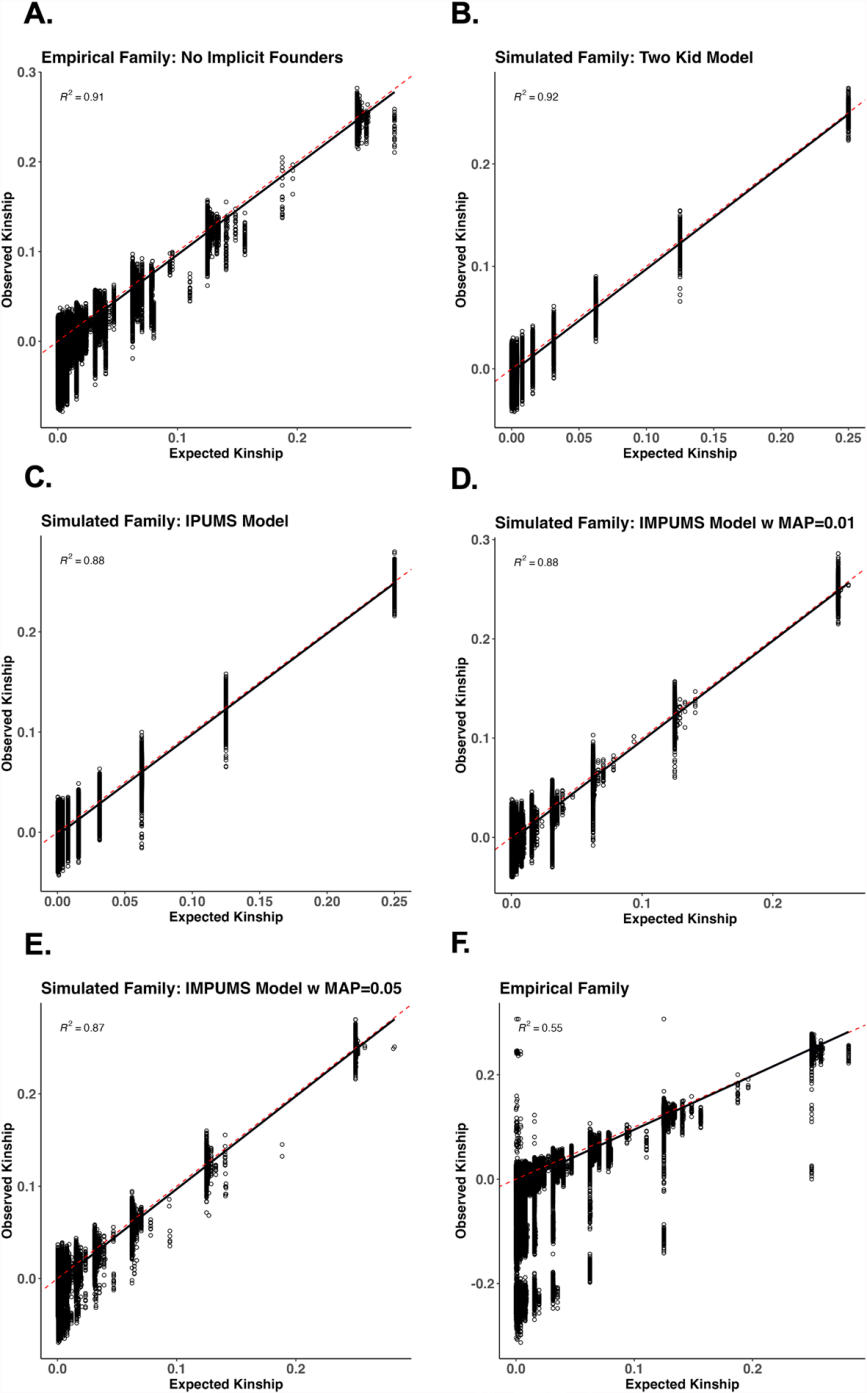
Scatter plots of estimated vs expected kinship across six different pedigree structures. The solid black line is a regression between estimated and expected kinship. The six different pedigrees generated represent (**A**) empirical pedigree from Familinx including implicit founders in the kinship estimates, (**B**) simulated family using a 2-kid model, (**C**) simulated family using census data for sibship distributions, (**D**, **E**) census simulated family with simulated misattributed paternity (MAP = 0.01, 0.05), and (**F**) empirical family from Familinx when the implicit founders are not included in the kinship estimate. The red dashed line represents a regression of the expected kinship across each relationship

Expected and observed kinship are strongly correlated for all the pedigree structures (Fig. 6). However, the empirical family had incomplete data, including offspring with only one known parent, thus including implicit founders (Fig. 6f). While our simulation framework allows the ability to simulate genomes for missing parents, the resulting VCF output does not contain the genomic profile of the implicit founders. This could introduce bias into the kinship estimate by not accounting for the absent parent's genetic contribution. Repeating the simulation by making all the founders in the pedigree explicit, the best line of fit improved in the empirical family (Fig. 6a), increasing the r^2^ value from 0.55 to 0.91 (Fig. 6 a and f). The rest of the families showed a high correlation between observed and expected kinships with r^2^ values ranging from 0.87 to 0.92. Overall, our results underscore the ability of py_ped_sim to simulate pedigrees with kinship estimates that align with expectations across various pedigree structures.

## Discussion

Py_ped_sim offers open-source tools for complex pedigree simulations and facilitates genome simulation onto pedigree structures using SLiM. We present a novel pedigree simulator that specifies distribution parameters for sibship size across generations, allowing for generational shifts in sibship rates. Additionally, by incorporating unexpected genetic half relationships into the simulation of family pedigrees, we further allow the modeling of dynamic pedigrees. This feature also adds the potential for consanguinity to be simulated in the pedigree. We offer the ability to simulate genomes based on user-provided pedigree structures via SLiM. py_ped_sim includes a wrapper for SLiM that includes automated identification of founders and assignment of generation numbers, saving users the cumbersome manual process with SLiM alone. Generation numbers are assigned in such a way to enable simulation of pedigrees with mating across generations and consanguinity. Users can create the starting genomes for founders, allowing them to simulate pedigrees from diverse populations and demographic histories. Finally, we also present a feature to identify genetic relationships between individuals within a family pedigree using three generalizable relationship metrics.

Our validation efforts demonstrate the software's proficiency in simulating genetic relationships with expected kinship levels by assessing the correlation between observed and expected kinship values in simulated pedigrees across various empirical and simulated pedigree structures. We additionally validate our simulation of pedigree structures, showing we can simulate non-uniform sibship rates of pedigrees, simulate misattributed paternity events, and extend the breath of an existing family pedigree based on user-provided parameters.

Despite these advancements, py_ped_sim has limitations, particularly with forward simulations, which can be computationally demanding when initializing founders with large genomes, even with SLiM’s performance optimizations. An important limitation of our pedigree simulator is the ability to simulate different rates of sibship for pairs of parents in the same generation if the user wants to use different sets of sibship distributions across different parts of the pedigree. This could be useful when different sets of parents within the same family have different rates of variations in the number of siblings. Another significant limitation is the lack of known half-sib relationships in our simulations. While our approach simulates half-genetic relationships by accounting for misattributed paternity, it does not simulate other scenarios involving half-genetic relationships.

Overall, py_ped_sim will help facilitate genomic analyses based on pedigrees across medical, evolutionary, and forensic sciences. While our primary focus on pedigree simulations centers on human kinship, this approach is adaptable to non-human organisms. This tool will allow simulations of large-scale ecological and evolutionary pedigrees, offering insight into inheritance patterns and understanding the evolutionary dynamics of populations. In forensics, py_ped_sim can be used to explore the performance of Investigative Genetic Genealogy over pedigree structures and genomic variation (Supplemental Figure S3). We additionally offer a feature to characterize genetic relationships by calculating three metrics from a DAG-represented family pedigree. Generalizing relationships between pairs of individuals within a family pedigree facilitates identifying connections across extensive family networks and streamlines the process of recognizing relationships across multiple pedigrees.

py_ped_sim offers an accessible solution for simulating pedigrees and genomes. It introduces the first simulation framework that incorporates real-world sibship size data into the modeling of pedigree structures. While other software may offer similar features for genomic simulations of pedigrees, py_ped_sim stands out for its flexibility, making it a valuable tool for researchers working with pedigree-based genomic data and potentially advancing further research in this area.

### Availability and requirements

Project name: py_ped_sim—A flexible forward genetic simulator for complex family pedigree analysis.

Project home page: https://github.com/MiguelGuardado/py_ped_sim.

Operating System:MacOS, Linux.

Programming Language: Python.

Other requirements: Conda for virtual environment dependencies.

License: GPL-3.0

Any restrictions to use by non-academics: None.

## Supplementary Information

Additional file 1

## Data Availability

Software can be found on Git Hub (https://github.com/MiguelGuardado/py_ped_sim). Empirical founder genomes used from 1000 genomes consortium can be found on their FTP site (https://www.internationalgenome.org/data/).
