# Supplementary material for: py_ped_sim: a flexible forward pedigree and genetic simulator for complex family pedigree analysis
Source: BMC Bioinformatics. 2025 May 7;26:122. doi: 10.1186/s12859-025-06142-z (PMC12060417; doi:10.1186/s12859-025-06142-z)
Supplement: Supplementary file 1 — Additional file 1 [file 12859_2025_6142_MOESM1_ESM.docx]

**Supplemental Text For:**

py_ped_sim - A flexible forward pedigree and genetic simulator for complex family pedigree analysis

**Authors**

Miguel Guardado^1,2,3,4*^, Cynthia Perez^5^, Sthen Campana^4,5^, Berenice Chavez Rojas^5^, Joaquín Magaña^5^, Shalom Jackson^5^**,** Emily Samperio^5^, Selena Hernandez^5^, Kaela Syas^1^, Ryan D. Hernandez^3^, Elena I. Zavala^5,6^, Rori V. Rohlfs^4,5*^.

1. San Francisco State University, Department of Mathematics, San Francisco CA, 94132, USA.
2. University of California San Francisco, Biological and Medical Informatics Graduate Program. San Francisco CA, 94158 USA.
3. Bioengineering and Therapeutic Sciences, University of California, San Francisco, San Francisco, CA; San Francisco, 94134, CA, USA.
4. University of Oregon; Department of Data Science; Eugene, OR, 97403, USA.
5. San Francisco State University, Department of Biology, San Francisco CA, 94132, USA.
6. University of California, Berkeley, Department of Molecular and Cell Biology, Berkeley, CA, 94720, USA.


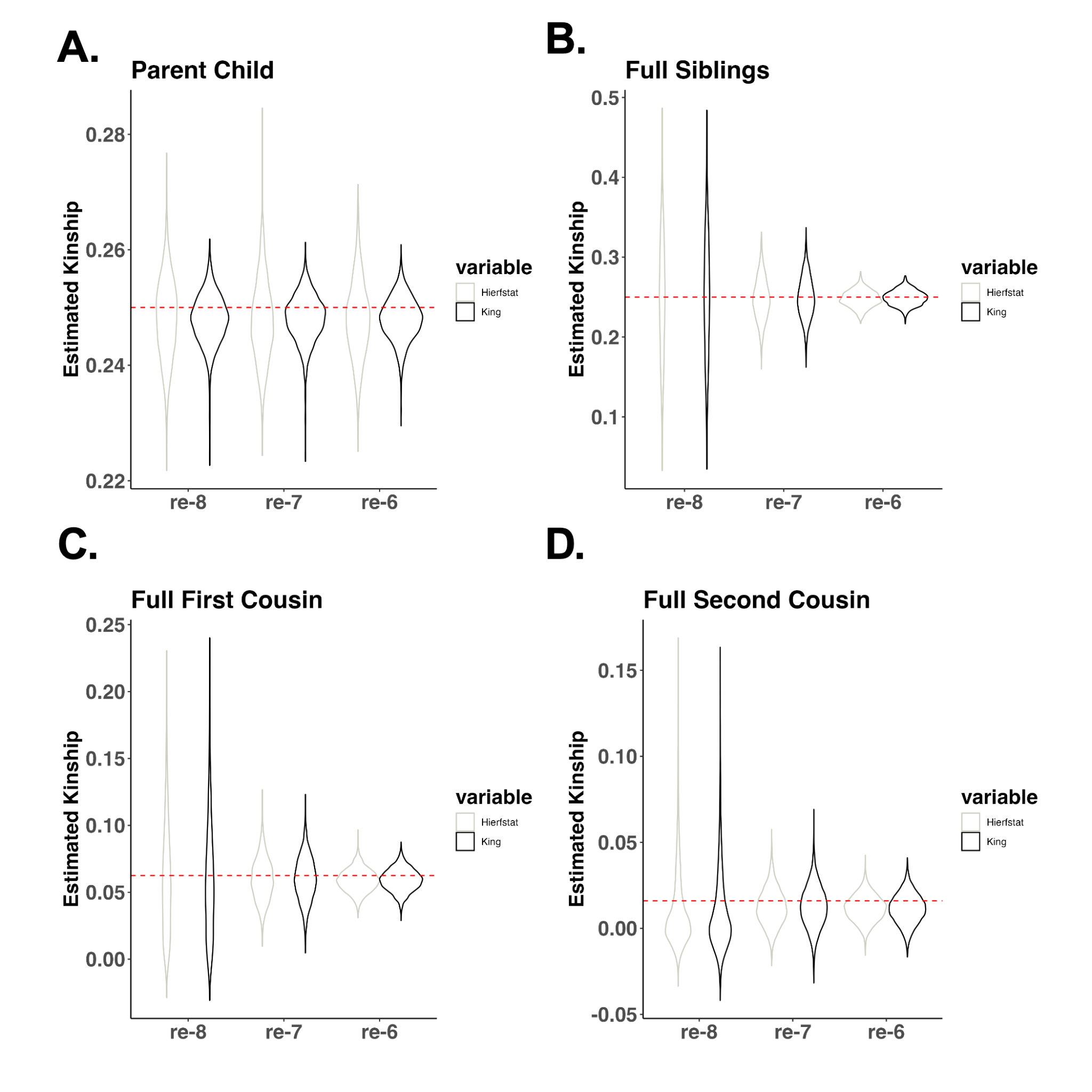


**Figure S1: Pairwise kinship estimates for a single family with varying recombination rates across four relationship types.** Four recombination rates are shown in ascending order, from the smallest to the largest. The red dashed line represents the expected pairwise kinship values for each of the four relationships. Pvalues (pval 1e-6,1e-7 = 0.33),

(pval 1e-6,1e-8 = 0.62), (pval 1e-7,1e-8 = 0.90).


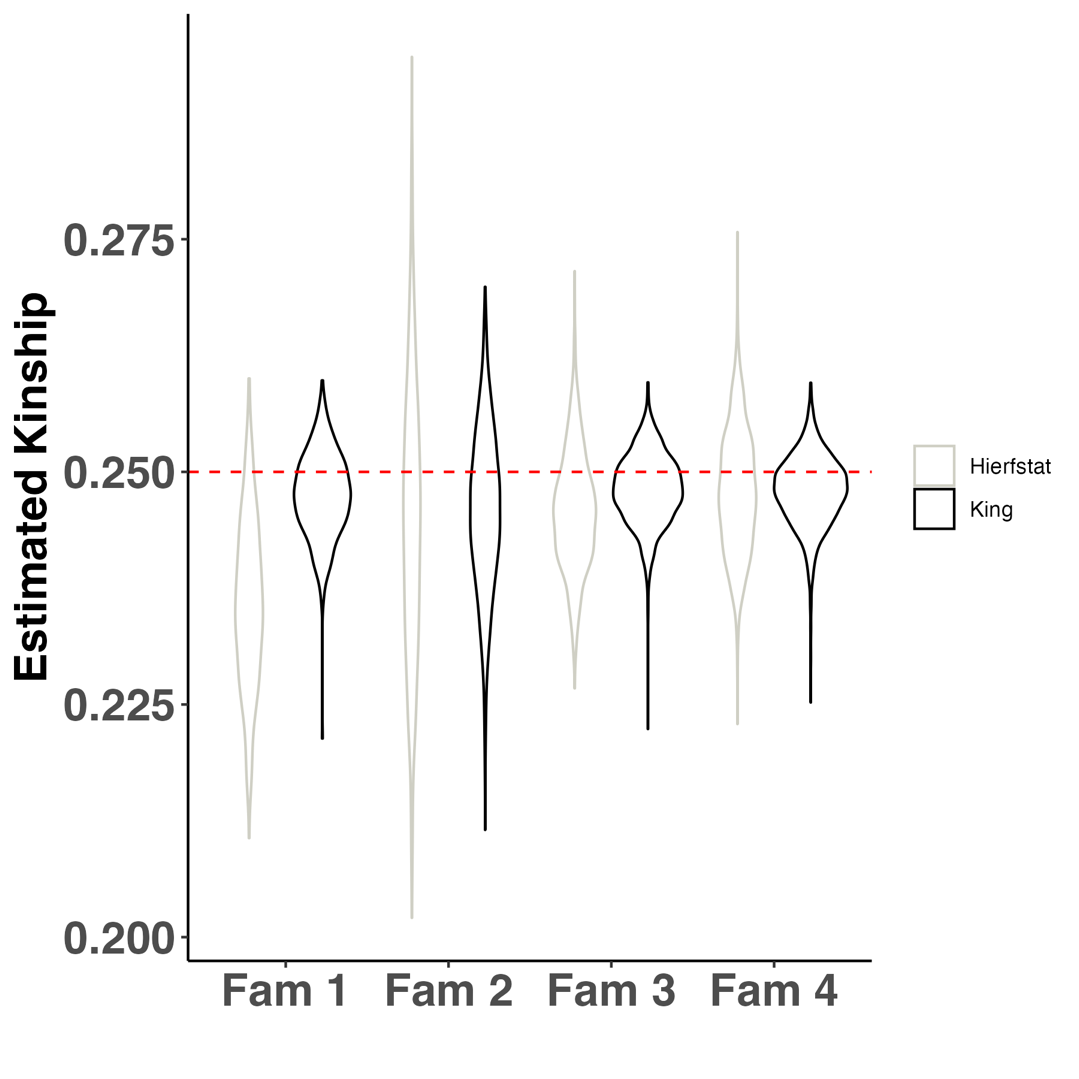


Figure S2: **Distributions of pairwise kinship estimates for parent-child relationships across four simulated families.** Genomes for the families were simulated using *py_ped_sim*. Kinship was estimated with two methods: KING (black) and hierfstat (gray). The red dashed line represents the expected kinship proportion for parent-child relationships.


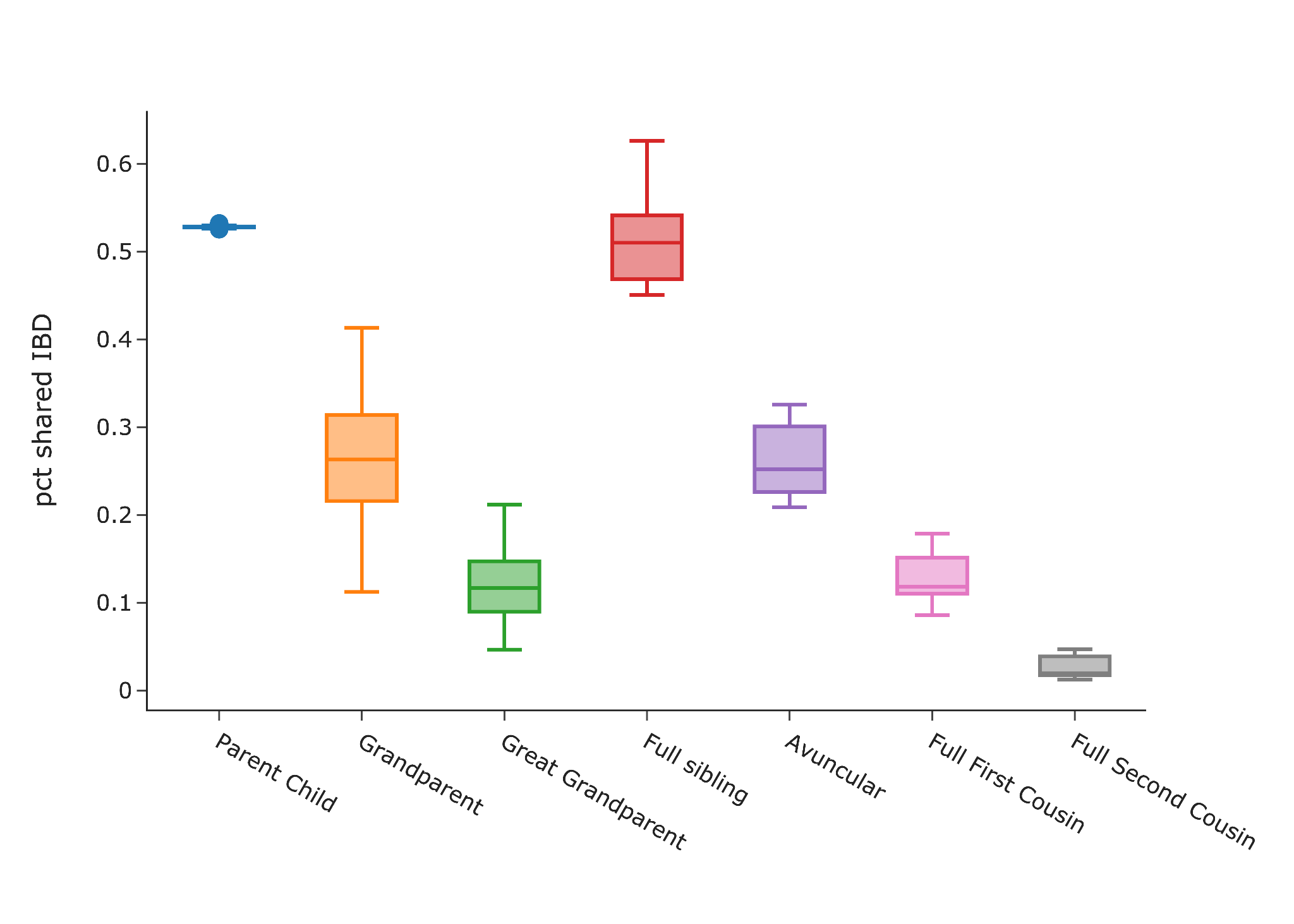


Figure S3: **Distribution of percentages of IBD shared for genomes simulated with recombination maps for seven genetic relationships**. Ten simulations were performed across a single family, genomes were simulated for 22 chromosomes. Pairwise IBD was estimated using hap-ibd.
